# Supplementary material for: Productive and reproductive performance of Dawuro highland sheep managed under community-based breeding program in Ethiopia
Source: PLoS One. 2023 May 11;18(5):e0284280. doi: 10.1371/journal.pone.0284280 (PMC10174558; doi:10.1371/journal.pone.0284280)
Supplement: Attachment — Submitted filename: Comment sheet -PONE-D-22-25221.docx [file pone.0284280.s001.docx]

**Review on**

**Productive and reproductive performance of highland Dawuro sheep managed under community-based breeding program in Ethiopia**

**Decision: Major revision**

I have carefully reviewed the manuscript number (PONE-D-22-25221) titled " Productive and reproductive performance of highland Dawuro sheep managed under community-based breeding program in Ethiopia”. After careful reading, I show great interest. The study aimed to evaluate the early growth and reproductive performance of Dawuro sheep managed under a community-based breeding program. The topic is very interesting and worthy of investigation. However, the manuscript needs the following major revision before publication. The detailed comments are as follows:

1) The abstract is needs to be improved. Author should report results precisely, add some quantitative results in abstract. At the end of the abstract, readers would like to see some practical policy proposal rather than a general policy statement. Please propose some solid policy implications derived solely from your own results.

2) The Introduction fails to motivate the study. In the present form, it resembles a mini-review of literature, rather than discussing any policy-level problem. Why this study is necessary? What policy level problem this study is addressing? How the study is expected to provide any solution to that problem? How does the choice of sample is complementing that problem? Are the results and policies generalizable? The introduction is silent in all these aspects. The author should improve introduction section and explain generic information.

3) Contribution of study is missing. Authors should report the contribution in the second last paragraph of the introduction section. I suggest selecting the most convincing single contribution and explaining and emphasizing it in more detail.

4) What is the aim of the review of literature? The authors have merely listed out the studies without even creating a debate among them. Without that debate and thoughtful contradictions, the research gap cannot be substantiated. There are inconsistency and lack of coherence between paragraphs. The sections/paragraphs should derive from one to another, particularly in sub-section of literature.

5) The literature review section is short; it is recommended that author should add more literature and add

6) The results are presented not in good order; however, some more in-depth discussion of your main findings would be fruitful. The author(s) need to compare their results (each finding) with past studies (what was provided in the article is not compares of results but an explanation of views from past authors) and in comparing the result from the empirical investigations the author(s) should as much as possible provide a recast of the comparison made and the supposed implications or advantages of the new finding made with those discovered by past authors. This will ensure justice to the extant literature and evincing the superiority of the current findings over the past findings.

7) Please include nomenclature of acronyms/abbreviations/symbols used in your manuscript and position it just after the last section.

8) The overall language of manuscript is not good. Author should need to improve the language of paper. In summary, after consideration of the above-mentioned concerns, the manuscript would be suggestable for publication.
